# Perspectives on Using Artificial Intelligence to Derive Social Determinants of Health Data From Medical Records in Canada: Large Multijurisdictional Qualitative Study

**DOI:** 10.2196/52244

**Published:** 2025-03-06

**Authors:** Victoria H Davis, Jinfan Rose Qiang, Itunuoluwa Adekoya MacCarthy, Dana Howse, Abigail Zita Seshie, Leanne Kosowan, Alannah Delahunty-Pike, Eunice Abaga, Jane Cooney, Marjeiry Robinson, Dorothy Senior, Alexander Zsager, Kris Aubrey-Bassler, Mandi Irwin, Lois A Jackson, Alan Katz, Emily Gard Marshall, Nazeem Muhajarine, Cory Neudorf, Stephanie Garies, Andrew D Pinto

**Affiliations:** 1 Department of Health Behavior and Health Equity School of Public Health University of Michigan–Ann Arbor Ann Arbor, MI United States; 2 Upstream Lab, MAP Centre for Urban Health Solutions Li Ka Shing Knowledge Institute Unity Health Toronto Toronto, ON Canada; 3 Primary Healthcare Research Unit Memorial University of Newfoundland and Labrador St. John's, NL Canada; 4 Department of Family Medicine, Rady Faculty of Health Sciences University of Manitoba Winnipeg, MB Canada; 5 Department of Family Medicine Dalhousie University Halifax, NS Canada; 6 School of Health and Human Performance Dalhousie University Halifax, NS Canada; 7 Department of Community Health & Epidemiology College of Medicine University of Saskatchewan Saskatoon, SK Canada; 8 Department of Family Medicine University of Calgary Calgary Canada; 9 Department of Family and Community Medicine, Faculty of Medicine University of Toronto Toronto, ON Canada; 10 Department of Family and Community Medicine St. Michael’s Hospital Toronto, ON Canada; 11 Dalla Lana School of Public Health University of Toronto Toronto, ON Canada

**Keywords:** artificial intelligence, social determinants of health, sociodemographic data, social needs, social care, primary care, machine learning, qualitative study

## Abstract

**Background:**

Data on the social determinants of health could be used to improve care, support quality improvement initiatives, and track progress toward health equity. However, this data collection is not widespread. Artificial intelligence (AI), specifically natural language processing and machine learning, could be used to derive social determinants of health data from electronic medical records. This could reduce the time and resources required to obtain social determinants of health data.

**Objective:**

This study aimed to understand perspectives of a diverse sample of Canadians on the use of AI to derive social determinants of health information from electronic medical record data, including benefits and concerns.

**Methods:**

Using a qualitative description approach, in-depth interviews were conducted with 195 participants purposefully recruited from Ontario, Newfoundland and Labrador, Manitoba, and Saskatchewan. Transcripts were analyzed using an inductive and deductive content analysis.

**Results:**

A total of 4 themes were identified. First, AI was described as the inevitable future, facilitating more efficient, accessible social determinants of health information and use in primary care. Second, participants expressed concerns about potential health care harms and a distrust in AI and public systems. Third, some participants indicated that AI could lead to a loss of the human touch in health care, emphasizing a preference for strong relationships with providers and individualized care. Fourth, participants described the critical importance of consent and the need for strong safeguards to protect patient data and trust.

**Conclusions:**

These findings provide important considerations for the use of AI in health care, and particularly when health care administrators and decision makers seek to derive social determinants of health data.

## Introduction

The social determinants of health (SDoH), people’s daily living and working conditions that are influenced by policies and structures (eg, racism and housing) [1], contribute to systemic and avoidable health inequities across groups [2,3]. These resource and power-related determinants contribute to access to high-quality health care services and delivery and widen the gap in health outcomes across sociocultural groups [4-6]. For example, people with lower-incomes in the United States often experience barriers to access to care due in part to gaps in health insurance, and many Black individuals have experienced health care discrimination and worse postoperative care outcomes than White individuals due to structural racism [7-10]. Income inequities persist in access to primary and specialist care in Canada despite universal health care [11,12].

Primary health care is at the nexus of medical care, public health, and community and social services [13-15], and the collection of SDoH data in primary care is essential to identify and tackle inequities, which contribute to poor health outcomes [4,16,17]. These data could be used to improve care, help patients with their social and financial situations through coordination to local services [17-20], and guide health care changes and public policy [17,19-21]. However, it is challenging to operationalize SDoH data in real-world clinical settings. Practical and technological challenges including a lack of a unified and standardized measurement of SDoH [22], electronic medical record (EMR) system variabilities [23-25], and finite health care system capacity, can lead to limited data interoperability and reduced power to inform health care systems and public health policies [23-25]. In addition, it is important to consider the health care team’s capacity with additional workload associated with administrating, collecting, documenting, and responding to needs, which lead to increasing risk of burnout [21,26-29].

Artificial intelligence (AI) can leverage the potential benefits of SDoH data to identify patient needs [30,31] and respond to health care overcapacity and disease complexity [32,33]. Machine learning is a common type of AI used to detect, predict, and categorize outcomes by looking for patterns in the data that are associated with known observations or “ground truth” cases [34,35]. Although this work is still in exploratory stages, machine learning and natural language processing have demonstrated the feasibility of detecting a number of SDoH from EMR data, such as childhood experiences [36], social connections [37], living situation [31,36], employment [31], and predicting health outcomes based on the SDoH [25].

While there is strong potential for adopting AI to identify SDoH data, there are multiple potential harms. Concerns with using AI technology in health care include the exacerbation of biases and inequities, discrimination [32], data security and privacy, and lack of infrastructure [38-40]. For example, the datasets themselves may reflect discriminative or biased practices, in which the algorithms are trained to learn [32,41]. Unstructured physician notes may contain biases which are similarly replicated to determine patterns and impact patient outcomes [32,41].

The perspectives of patients and the general public (who have been or may become patients) are critical to shaping decisions surrounding the implementation of AI in health care. Patient data are used to develop AI algorithms and patients are affected by having AI inform their care [42]. Despite its importance, few articles focus on public or patient perspectives on AI in health care [38-40,42]. A scoping review of 37 articles (grey literature and peer-reviewed) found that many patients reported positive views on AI, although views may differ based on patients’ experiences, concerns, and trust [42]. However, there is limited knowledge of participants’ perspectives on using AI to derive their SDoH information using existing EMR data, which may elicit different views due to the sensitive nature of this information. Given the widespread adoption of EMRs in primary care settings, the sensitivity of SDoH data, and the potential harms associated with AI in health care, it is essential to learn viewpoints on whether and how AI could be implemented in an equitable, acceptable, and safe manner. This qualitative study aimed to understand the perspectives of a diverse sample of Canadians on using AI to derive SDoH information from existing EMR data in primary care, including potential benefits and concerns.

## Methods

### Study Background

This study was conducted as part of a multicomponent project that developed and refined a standardized SDoH questionnaire for primary care settings, known as the Screening for Poverty And Related Social Determinants and Intervening to Improve Knowledge of and Links to Resources (SPARK) Tool (Multimedia Appendix 1) [43]. Details on the SPARK Tool and the broader project are provided elsewhere [43]. For this paper, we report on data gathered from in-depth interviews with participants related to their perspectives on having AI derive SDoH data from the EMR. Participants became aware of the SDoH before in-depth interviews by completing the SPARK Tool and were provided with examples of determinants.

### Study Design, Setting, and Sampling Approach

A qualitative description approach was chosen to provide a comprehensive summary of participants’ experiences and preferences on using AI to derive SDoH data in primary care settings [44]. One-on-one, semistructured interviews were conducted using video teleconference software across 4 Canadian provinces: Ontario, Newfoundland and Labrador, Saskatchewan, and Manitoba.

Maximum variation sampling helped to ensure that a diverse sample of participants were included across ages, races and ethnicities, location (rural or city), languages, and gender identities where possible [45]. After indicating interest in the study, potential participants were asked about these characteristics, which enabled the purposive selection of individuals who were not initially well-represented in the sample. Adults (aged 18 years and older) were recruited through social media advertisements, email distribution lists, and posters in community and health centers [43]. Advertisements were translated based on the 3 most commonly spoken languages in each province other than English and French, although interviews were only conducted in English due to a lack of non-English speakers contacting the study team. The study aimed to recruit a sample of approximately 200 individuals, with a greater number of participants from Ontario due to the size of the province, to ensure adequate diversity across the multiple domains of the SPARK Tool and to inform the multicomponent study objectives and sampling approach.

### Data Collection

The interviewers were female research staff [DH, AD-P, AZS, LK, and IAM] or research assistants, and all received training or had previous experience with qualitative interviewing. The study was also informed regularly through a national advisory group composed of patient partners, scientists, and other collaborators. Data collection occurred from April 2021 to January 2022. The interview guide was established and iteratively revised through meetings with the research team and participant feedback to ensure that the questions were comprehensible for participants without a background in AI. Participants provided verbal informed consent before participating in the study, in accordance with research ethics board requirements. Participants were asked their perspectives on having secure software access patients’ records to derive their SDoH information, as opposed to asking patients for this information directly (refer to Multimedia Appendix 2 for interview questions). Follow-up questions included examining possible benefits or concerns with the use of this software.

### Data Analysis

Interviews were audio-recorded and transcribed verbatim using a professional service. NVivo (version 12; Lumivero) software was used for data management. The codebook was developed collaboratively by the interviewers and the analysis team (VHD, JRQ, IAM, DH, AZS, LK, AD-P, and ADP). Two study team members (VHD and JRQ) with qualitative research experience led the analysis. An initial codebook outline was created by 2 of the interviewers (AD-P and IAM) during independent review of 2 transcripts. This outline helped to guide the development of a preliminary codebook, which was developed by the lead analysts after examining 10 randomly selected transcripts, including the 2 previously examined transcripts (4 from Ontario, 2 each from Newfoundland and Labrador, Saskatchewan, and Manitoba). The remaining analysis team members reviewed the same 10 transcripts and provided detailed feedback on the preliminary codebook. After the codebook was revised and agreed upon, 2 study team members continued to refine it by reviewing 2 additional transcripts each. Codes were compared for consistency and alignment, and extensive documentation was created to outline decisions made during multiple meetings between the coders. The codebook continued to undergo minor edits as more transcripts were reviewed. After 12 transcripts were thoroughly reviewed and examined between the 2 coders, the remaining interviews were randomly split within each province between the coders.

A qualitative content analysis was conducted to focus on theme development across participant interviews [46-48]. A combined inductive and deductive approach was used to enable flexibility to incorporate emerging codes and findings from transcripts, while also focusing on codes pertaining to the research and interview questions.

**Figure 1:**
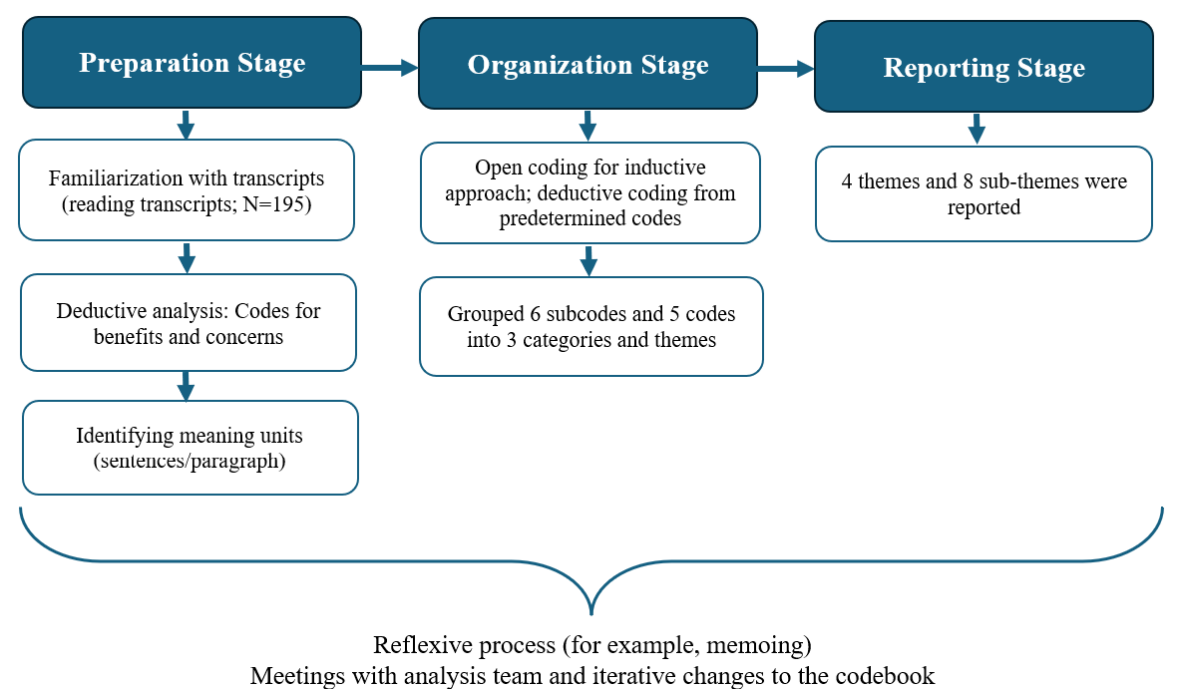
Qualitative content analysis based on the methodology by Elo and Kyngäs [48].

The researchers familiarized themselves with the transcripts before analysis by thoroughly reading the contents. For the inductive coding approach, the transcripts were read and meaning units (chunks of data, such as sentences or a paragraph) were selected and coded without a predetermined plan [46,47]. For the deductive coding approach, codes specific to potential benefits, challenges or concerns with using AI to derive SDoH information were created before analyzing the data based on the research aims and interview questions. Meaning units were assigned to these predetermined codes, and codes were grouped into categories and themes in an iterative process. Throughout the data analysis, regular meetings were scheduled with the analysis team to discuss updates, preliminary findings, and assist with the context and interpretation of codes into themes. Figure 1 provides a simplified description of the analysis workflow based on the methodology by Elo and Kyngäs [48].

### Positionality, Reflexivity, and Trustworthiness

The 5 main interviewers (IA, DH, AZS, LK, and AD-P) reside in different provinces and have different backgrounds and perspectives. The team regularly met to discuss the interviews and the project both while the interviews were being conducted and when the analysis was occurring, alongside VHD, JRQ, ADP, and at times EA. This team approach helped to mitigate any 1 perspective from dominating the interviews and analysis, as a form of data triangulation to enhance the criteria of trustworthiness [49].

The 2 main analysts (VHD and JRQ) engaged in reflexivity to understand how their own biases and preconceptions could influence how the data were approached and analyzed, through regular journaling and discussions before and throughout the analysis [49,50]. They also took memos upon analyzing interviews to document early findings [49,50]. For example, they documented their own beliefs about whether AI should be used to derive SDoH information, which became more nuanced based on the benefits and concerns expressed by participants. Both analysts created extensive documentation outlining the processes and decisions for the coding approach and analysis [49].

### Ethical Considerations

This study was approved by the Unity Health Toronto Research Ethics Board (#20-241) Saskatchewan Behavioural Research Ethics Board (#2373), Newfoundland and Labrador Health Research Ethics Board (#2020.259), and University of Manitoba Health Research Ethics Board (#HS24204).

## Results

### Overview

There were 195 interviews conducted across the 4 provinces, lasting approximately 30-45 minutes each. Most participants lived in Ontario (124/195, 64%), identified as women (126/195, 65%), and non-White (122/169, 72% among those who disclosed their race and ethnicity), while 38% (75/195) of participants reported at least 1 unmet social need (Table 1).

**Table 1:** Participant demographics (N=195; adapted from Adekoya et al [43], which is published under Creative Commons Attribution 4.0 International License [51]).

Characteristics			Participants, n (%)^a^
**Canadian province**			
	Ontario	125 (64)	
	Saskatchewan	25 (13)	
	Manitoba	24 (12)	
	Newfoundland and Labrador	21 (11)	
**Race and ethnicity**			
	Asian	71 (36)	
	Black	17 (9)	
	Indigenous	8 (4)	
	White	47 (24)	
	Other	10 (5)	
	Multiracial	16 (8)	
	Data not collected (Manitoba) or no response^b^	26 (12)	
**Gender**			
	Man	58 (30)	
	Woman	126 (65)	
	Transgender, gender fluid, or nonbinary	9 (5)	
	No response	≤5	
**Sex at birth**			
	Male	58 (30)	
	Female	133 (68)	
	Intersex	≤5	
	No response	≤5	
**One or more unmet social needs^c^**			
	Yes	75 (38)	
	No	120 (62)	
**Difficulty making ends meet**			
	Yes	43 (22)	
	No	151 (77)	
	No response	≤5	
**Highest level of educational attainment**			
	Less than a high school diploma	≤5	
	High school diploma or some postsecondary education	48 (25)	
	Trades certificate or diploma	14 (7)	
	College or university degree	94 (48)	
	Postgraduate degree	34 (17)	
	No response	≤ 5	

^a^Percentage values are not provided for values that are ≤5.

^b^Race and ethnicity data were not collected in Manitoba.

^c^Unmet social needs were based on the following categories: precarious employment (presence of all of the following: short term, casual, or temporary employment; fear of being fired if raised employment concerns; and varying pay); living in social housing; missed rent or utility bill payments; missed appointment due to transportation cost; avoided filling prescription or made it last longer due to cost; difficulty making ends meet; and lack of social support.

### Themes

The results are presented as main themes and subthemes. The 4 main themes include AI as the inevitable future; potential health care harms; loss of the human touch; and consent is critical. Additional quotes that provide evidence of themes are provided in Multimedia Appendix 3.

#### AI as the Inevitable Future: Facilitating More Efficient, Accessible SDoH Information and Use

Participants described common benefits to leveraging the use of AI in medicine to derive their SDoH information, as it represented the future of technological advancements, and “it’s much better that way” (05_20, Newfoundland and Labrador). Some participants spoke of AI as inevitable regardless of their perspectives for or against its use and seemed to acquiesce to its use in medicine.

Yeah, I’m for it… I mean it’s the future of medicine and it’s the future of the world so… Whether I have concerns or not, it’s going to take over, but personally I don’t have concerns, I’m okay with it, yep.

01_135 (Ontario)

##### Efficient, Streamlined Social Determinants of Health Data

Most participants expressed that the benefits of using AI in primary care were to streamline SDoH data collection in a more efficient and timely manner for patients, staff, and the health care system.

Well, I see benefits cause it streamlines the process and makes your information more accessible to people so you’re not the one who has to like repeat and remind [healthcare staff] all the time.

04_16 (Manitoba)

One participant mentioned that, if used correctly and accurately, it could reduce the burden on the health care system to free-up resources for providers to speak with patients.

People think that artificial intelligence and technology will be replacing jobs but like that’s not true because it only is there to help us move faster in life…Once you give the job [of deriving patients’ SDoH data through AI] …the people who were doing that [previously] could have one-on-one…interpersonal communication with patients and you know speak to them while they’re in the waiting room.

01_06 (Ontario)

##### AI Could Overcome Barriers to Disclosure

A small number of participants described how using AI to derive SDoH information based on existing EMR data could overcome barriers to verbally disclosure, in a “more accessible” (01_124, Ontario) manner.

So, I do [see benefits of AI], I think like folks that have challenges expressing themselves like…people that are vulnerable…the homeless, folks that are coming out of traumatic situations…people that have language barriers, speech barriers; I think this technology would be very beneficial to them…And people that have disabilities.

01_99 (Ontario)

##### Data Use to Improve Health

Participants expressed ways that using AI to derive SDoH information could be useful to improving health or health care. For example, 1 participant described how it would help to

…*move towards a system of greater continuity of care and a system where you are not forced to tell each new professional you see the same old story*. [01_134 (Ontario)]

Participants expressed that AI could help generate automated alerts of potential conditions for individuals based on group memberships from the SDoH data, in addition to other health information in the EMR.

I think it’s good for certain things like I think if you’re… from a particular country or race or something and you’re prone to very like high health risk diseases for example…I think that’s important and sure a computer can fill that information out so that way they red flag like check her heart every time she comes in.

01_68 (Ontario)

Participants indicated that AI could be useful to help automate local social and community resources to assist with their social situation.

That’s a way to screen and actually find the best help for you within your area…say for example the person has addiction or…mental health challenges or…are in an abusive situation…Sometimes these programs are so filled there is not enough space available…this program can provide all this information accessible to you and show the patient immediately if tomorrow they can get help… they’re put on some waiting list or [indicate where] there are shelters available or…programs from the government.

01_38 (Ontario)

Some participants expressed that disaggregated SDoH data could be used at a larger health care or population level, as opposed to the individual level. This included assistance with policy-making decisions to reduce health inequities, disease surveillance, understanding the causes of diseases, and prevention. COVID-19 disparities were mentioned as an example of the use of SDoH data collection.

I think the benefits is if we’re going to be using to track down diseases like not [individual] people [and determine]…What are the cause?... That would be beneficial and [it could be used for prevention] … [You could] do more campaigns…[but] knowing the stats and releasing that to the public in general I think that would help everybody…[It would help] to make them aware of what’s going on… Especially now with COVID.

03_16 (Saskatchewan)

Similarly, 1 participant described how the information generated could be used to inform “culturally competent” care through staff training practices.

You [can use AI for demographic purposes, such as you] find that you have 50% African, 30% Asian, 10% White, 20% mixed [racial identities] … Then it would therefore affect training because clearly the majority of your clientele will be African so you have to be culturally competent to be able to treat [them] with whatever they are coming with, the possibility so I think in that sense it could be beneficial.

03_19 (Saskatchewan)

#### Potential Health Care Harms: Distrust in AI Used in Public Systems

Many participants held strong views about the potential harms of implementing AI in primary care and expressed an overall distrust in AI.

##### AI Inaccuracies and Impact on Care

The primary concerns with using AI to derive SDoH information in health care were inaccurate SDoH predictions and the subsequent consequences on their care provision. Many participants were particularly concerned about their race and ethnicity, gender identity, and sexual orientation being incorrectly identified, including “misclassifications” (01_28, Ontario).

I think that could actually kind of be dangerous… I’m not sure I would trust the computer…I mean I work in [information technology] but [Laugh] I actually wonder how would a computer know for sure what my racial identity is?... I’m just afraid of the computer making the wrong choice and then it somehow impacting my healthcare negatively… If the computer [was] in the background [and] was only doing it for statistics that is not for an individual’s healthcare then I mean I don’t really care. I feel like there could be room for error.

01_32 (Ontario)

However, a few participants felt that more inaccuracies would result from a human compared with AI and they would trust the computer over humans with their SDoH information:

I think maybe a computer would probably be better than an individual doing it because you know [for an] individual there is always I would think a larger chance for a margin of human error rather than a computer.

01_56 (Ontario)

With regards to updating SDoH information, some participants expressed that social situations are malleable and may change, but those changes may not be accurately captured in their EMR by their physician unless they are explicitly asked about it through a survey. Participants mentioned this often in the context of financial situations, sexual orientation, or gender identity.

I think asking people one-by-one even though it is a bit more time consuming is worth it because you know like even for sexuality and all like it always changes so it’s not like they can, it can be generated once because it should come from the main source…because like let’s say you’re in a court and someone says like you know where did you get this information from? It’s like I didn’t even provide that information.

01_39 (Ontario)

It was described how AI would not pick up on the subtle human cues that are intrinsic to conversation.

The only problem, when it comes to like using that type of technology is there’s no emotion behind it so when you’re speaking with someone one-on-one you might say something in a certain way but you don’t mean it that way. So, your facial expression, the way you speak it communicates your idea across differently.

01_27 (Ontario)

In addition, some participants had different perspectives on using AI based on the SDoH variable that was predicted. They mentioned that perhaps certain SDoH should be derived using EMR data while others should be asked directly by patients.

So, I think for things that are like you know pretty like core defining questions like your race, your gender, your age, your citizenship like I don’t want the thing guessing that I’m Chinese based on my last name for example…So, I think those things are, you know like more like the legal aspects of things… Other things like you know your income like okay, it can predict it and probably it’s like pretty accurate…I would just want to be assured that whatever predictions it makes like it would be to try to benefit me and not to try to like characterize myself and like sell me some new drugs.

01_72 (Ontario)

##### Privacy and Security

Privacy and security were important concerns of participants:

I think that privacy…has to be the number one issue that guides every aspect of this.

04_21 (Manitoba)

Participants expressed concerns with the management of SDoH information in the EMR, including that “…*the infrastructure and the environment in which you are operating is not equipped to protect the data you are collecting*.” (01_13, Ontario). Another participant mentioned “*It’s frightening what computers are being programmed to do without any accountability.*” [04_21 (Manitoba)].

Some participants also mentioned that the data being retrieved using EMR was in itself a “huge invasion of privacy” [01_80 (Ontario)].

Several participants described how they did not want AI to derive their SDoH information as it removed their ability to control and share what they are comfortable with sharing to their doctor, given the sensitive nature of the SDoH questions. For example, some participants were themselves not willing to have their SDoH data on the EMR, such as their race and ethnicity or sexual orientation.

Importantly I think it would make me very leery or weary of what I share every time I talk to a healthcare provider cause I might want to share a sensitive piece of information but I don’t want that information to be widely available on my record …Your program to retroactively go back and gather this information from all of my visits just makes me feel very like not in control of my own information.

01_50 (Ontario)

The interviews occurred at a time when there was media attention surrounding hacking and privacy breaches of government across provinces, and COVID-19 vaccine policies. Individuals also reported having distrust in government and overall technology.

My thoughts on this whole COVID-19, yeah, it’s a long story and stuff but I don’t trust the government so I wouldn’t want it to be done... I don’t have a lot of trust in computers... You can say everything is secure but like I said I’ve seen it over time and especially in the last 20 months there’s been a lot of breaches…You want to know? Phone me. You want to question me or ask me? Bring me in person-to-person. Don’t do it over the computer... because you can be hacked at any time.

03_22 (Saskatchewan)

##### Data Misuse and Discrimination

Few participants mentioned their concerns about data misuse and discrimination. For example, a participant mentioned how the use of AI would be stigmatizing and could negatively stereotype people, particularly those living with disabilities. Discrimination due to race and ethnicity and income were also reported by participants.

Some participants extrapolated the harms beyond the health care system. Participants compared the potential for AI to discriminate or be used for discrimination, to their concerns regarding the criminal system and policing. For example, 1 participant indicated that it would facilitate “[racial] profiling in general and not only healthcare but the government is going to use it, so I am concerned” and provided an example of it being used in fascist governments for “genocide against communities” [01_07 (Ontario)].

There were also concerns regarding the data being misused if it were to be leaked.

The companies are trying to surveil us or they’re trying to… use our data for their own means…I’m thinking about like police officers... I think it was in British Columbia where like…the municipal police department …was using AI to surveil folks and… that is like very problematic and could really harm a lot of people and I think that this could just be linked to that and like I feel like police would maybe somehow have access to this if the government does.

01_41 (Ontario)

One participant described how the algorithm would essentially be stereotyping individuals in order to predict and identify their SDoH information.

Well, and it’s based on a whole lot of basically stereotyping groups so saying oh, well I am racialized and low-income therefore these things must be true and I’m not at all comfortable with, cause it’s, the whole system relies on those stereotypes and assumptions.

04_17 (Manitoba)

However, 1 participant believed that AI “is not racist. [Laugh] They don’t see race” [03_24 (Saskatchewan)] and is better suited to SDoH data retrieval as opposed to human data collection.

#### Loss of the Human Touch: Preference for Provider Relationships and Individualized Care

A few participants expressed an overall preference for having strong patient-provider relationships and interactions and emphasized the need for individualized care. Participants believed AI could pose a barrier to patient-centered care and the patient-provider relationship, by removing the “human factor” with providers which could otherwise be nurtured by having a conversation about their SDoH situation. For example, they expressed that it would be “de-personalizing encounters with the doctor” [04_20 (Manitoba)] or would be “dehumanizing,” particularly for people experiencing a mental health condition and social isolation, by “just being dealt with by a machine” [01_125 (Ontario)].

I think having the human touch to our view like these surveys is always good because well people will give more personal opinions if you allow them to whereas if it was just AI then you know we’re just numbers.

01_36 (Ontario)

Healthcare should be about individuals and it should be about connection. It should be about you and me face-to-face and to put artificial intelligence in there to pigeon hole me into a certain group I think would be very dangerous. I think we want to get away from that and start looking at individual situations and everybody’s you know life and how we can interact with them best.

03_03 (Saskatchewan)

Similarly, one participant provided a unique perspective as someone who identifies as living with disabilities.

I’d just like to see that be individualized and person centered... That requirement for person-centered care for diversity of options, a diversity of supports, and for that holistic care to be present and connected and supportive. Mostly I think it’s a human rights actual systemic problem to use AI.

03_21 (Saskatchewan)

#### Consent Is Critical: Strong Safeguards Are Needed to Protect Patients’ Data and Trust

##### Overview

Many participants advocated for strong safeguards and fully-informed consent before having AI used to derive their SDoH information. Most of these participants described opt-in consent, however some described opt-out consent. There is a need for “full transparency” [01_78 (Ontario)] regarding why the data is being collected by AI, what it will be used for, how it will be stored, who has access, how it will work, and oversight before implementation. Without these safeguards, participants mentioned that it would be a “breach of trust” [01_78 (Ontario)] and they would feel upset and “caught off guard” [01_38 (Ontario)].

And I think verbal consent or written consent is very important…as opposed to the passive consent you know it’s like when you download something how it’s all agree, agree, agree…We really do need to read the fine print…And because I think that we, our private information is more valuable than we actually realize and we’ve gotten so accustomed to passing it out at times that we’re failing to realize how valuable and how sensitive it can be so….

05_12 (Newfoundland and Labrador)

So it is a black box in the way it uses that [information] but then is there a team which is always correcting it if it’s out of whack?

01_13 (Ontario)

##### Data Access and Verification

Multiple participants wanted the opportunity to check what the AI algorithms derived for their SDoH information and have the chance to rectify inaccuracies.

Maybe you know it would be okay if the AI [derives the SDoH information] first and then the patient…is asked to confirm like say here is what we think it is, do you think this is correct - and then the patient can say yes or no but I think not to just do it and like keep it there behind closed doors.

03_05 (Saskatchewan)

## Discussion

### Principal Findings

Overall, there were varied perspectives on whether AI should be used in primary care to derive patients’ SDoH information. Some participants expressed that the benefits of more efficient data collection or improved care did not outweigh the potential harms from inaccurate predictions, privacy and security concerns, reduced patient-provider interactions, and data misuse and discrimination. Many participants emphasized the need for strong safeguards if AI is used, including fully informed consent, transparency, oversight, and the ability to check and verify predictions.

#### Benefits

Participants in other studies have similarly identified that the use of AI in health care is efficient and could promote improved health and health care [52]. Integrating SDoH information through AI can help to predict risks of negative health outcomes (eg, suicide [53] and HIV [54]) and health care use [25,55-57]. Other studies have found that the identification of unmet social and financial needs using EMR data could assist with predicting the need for community resources, and therefore facilitate community resource connections and personalized interventions to address patients’ social situations [57,58]. It could also help to inform future program design and community planning to build capacity and outreach to encourage access to necessary health services and reduce related inequities [57,58].

#### Navigating Concerns and Establishing Strong Patient Protections

Many of the potential harms of AI were focused on issues surrounding participants’ personal care and treatment at the individual-level, as opposed to the use of aggregated data for system- or organization-level change. This may partly explain the strong concerns that surfaced about AI. Some participants highlighted the impracticality of continuously verifying AI-generated outcomes over time and expressed willingness to contribute their data anonymously for research and quality improvement. Health services may consider the feasibility of adopting alternative models of patient consent [59]. For example, tiered consent enables patients to opt-in or -out of sharing their data under various conditions (eg, data used at the aggregate level for algorithm development or individual-level) [59,60]. It has been recommended for organizations to implement patient education on consent regarding AI and to work alongside patients in creating a consent that is coherent and straightforward [60]. Similar to the participants in this study, others have discussed consent, transparency, verification, control, and oversight as part of recommendations for the ethical use of AI [55,61-64].

AI is a broad term to describe many types of complex statistical methods, which are not often transparent or well understood by the public. This can lead to mistrust in AI and medicine, and given the novelty of AI in health care settings, there are still many unknowns. A major concern is the potential for AI to misclassify one’s racial or ethnic background, as well as fluctuating social circumstances. Even with highly accurate AI models, misclassification is possible, as well as algorithmic or data bias and societal bias [32]. Without careful oversight, inaccurate or biased AI models could be used to significantly impact patient care or propagate discrimination or health inequities [32,41]. This may contribute to overcriminalization and systemic discrimination experienced by racialized and low-income communities [65,66]. Thus, although resource-intensive, it is imperative that patients have the ability to verify the AI-generated SDoH outcomes and modify it. It is also incumbent on AI teams to produce algorithms that go further than just being interpretable, but ones that provide justification for the outcome [67] and proactively address health equity [68].

Other studies examining perspectives on AI in health care have reported similar concerns of privacy and data breaches, infrastructure and oversight, and lack of choice or control of the data [24,38,39,52,62,63,69]. During the time of the interviews, the Canadian news media frequently discussed ransomware attacks and data breaches in medical data systems [70], including those described as the “worst in Canadian history” [71]. This context could have impacted participant concerns of using AI to derive SDoH data in this study.

Health care, particularly primary care, is a highly personal “social enterprise, powered by committed, caring, and collaborative connections between the humans involved” [72]. It is not surprising that a major concern in this study was the potential for AI to threaten patient-centered and individualized care, harming the patient-provider relationship. These findings have been reported in the literature [69,73] and may reflect concerns about AI in medicine in general, as opposed to extraction of SDoH data on the backend. Some articles have suggested restricting AI from decision-making [74,75] such as automatically deriving patient SDoH data and recording it into patient records. Instead, AI could be used as a tool [74] as part of patient-centered care that helps providers prioritize discussions about SDoH with their patient based on their risk of having unmet needs. Based on these discussions, team-based care approaches could support personalized actions following identification of a need.

Implementation of AI for SDoH data extraction should incorporate a strong focus on equity and meaningful partnerships with underrepresented communities in every stage, to promote safety and minimize potential harms [32,38,62]. This should include community governance, particularly for Indigenous and Black communities [61], and would inform whether the SDoH data is collected, the type of data to collect, the frequency for such data collection, and its use. In combination, using an equity framework to guide implementation could help to mitigate medical mistrust and associated health disparities [76,77], particularly among individuals who experience structural racism and discrimination in health care [76,78]. Overall, while the necessary safeguards may diminish some of the returns of more timely, efficient SDoH information retrieval using AI, they must be an inextricable part of using AI to derive SDoH data.

### Strengths and Limitations

This study has multiple strengths. It involved a large sample of 195 participants across 4 Canadian provinces and used a maximum variation approach for a diverse sample. For example, the majority of participants who disclosed their race and ethnicity identified as non-White, and most had at least 1 unmet social need. There are few multijurisdictional studies that examine perspectives on using AI to derive SDoH data; thus, this study contributes to reducing knowledge gaps regarding implementation of AI to derive SDoH data in primary care. Various techniques were also used to increase the trustworthiness and quality of the study. Furthermore, the SPARK project is composed of a dedicated team of researchers and patient partners who are passionate about health and social inequities and meet regularly to discuss the study and decision making.

Several limitations were also identified. This study reports the findings from questions on AI that were asked as part of a larger interview on general SDoH data collection and use in primary care. Participants’ perspectives may have been impacted by the other questions that preceded the AI conversation. In addition, despite having study recruitment materials available in multiple languages for each province, the team relied entirely on potential participants to make initial contact and were not approached by non-English speakers. While the sampling strategy captured a range of formal educational experiences, approximately one-third had a college, university, or postgraduate degree. Thus, the study sample may not include individuals who experience language barriers and may be less transferable to individuals who did not complete high or grade school. Furthermore, race-based data in Manitoba were not collected due to the need for greater community engagement. This study did not receive Research Ethics Board approval across all provinces to report participants’ age; however, each province considered age for a diverse recruitment sample. Finally, given project timelines and resource constraints, qualitative data analysis was not formally initiated until the interviews were completed. Despite this, the close communication, regular meetings, and feedback enabled the interview guide to undergo changes for greater clarity; the interview questions were also piloted by the study team beforehand.

### Conclusions

This large qualitative study examined perspectives on using AI to derive SDoH data in primary care using existing EMR data. Participants described the benefits of efficiency, access and improvement of care, and concerns focused on inaccuracies, negative consequences to care, privacy and security breaches, reduced patient-provider interactions, data misuse, and discrimination. Strong safeguards, including fully informed consent, verification and oversight can help to alleviate harm. There is a need to engage communities in the development, implementation, and evaluation of future AI initiatives, to determine opportunities and safety measures for using AI. Interviews with providers, health care administrators, and decision-makers are also necessary to understand the feasibility of integrating AI in primary care for SDoH information.

## Appendix

Multimedia Appendix 1 SPARK (Screening for Poverty And Related Social Determinants and Intervening to Improve Knowledge of and Links to Resources) tool.

Multimedia Appendix 2 Interview questions assessing sociodemographic data Collection using artificial intelligence.

Multimedia Appendix 3 Additional representative quotes.

## Abbreviations

AI: artificial intelligence
EMR: electronic medical record
SDoH: social determinants of health
SPARK: Screening for Poverty And Related Social Determinants and Intervening to Improve Knowledge of and Links to Resources
