# Supplementary material for: Perspectives on Using Artificial Intelligence to Derive Social Determinants of Health Data From Medical Records in Canada: Large Multijurisdictional Qualitative Study
Source: J Med Internet Res. 2025 Mar 6;27:e52244. doi: 10.2196/52244 (PMC11926464; doi:10.2196/52244)
Supplement: Multimedia Appendix 1 [file jmir_v27i1e52244_app1.docx]

**Appendix 1. SPARK TOOL**

*The SPARK Tool has been published in appendices elsewhere, including the Journal of General Internal Medicine,*^1^ *PLOS ONE,*^2^ *and as a pre-print on medRxiv.*^3^

**Sociodemographics**

1. **If it could be arranged, would translation into another language be helpful at your next appointment?**
   - Yes
   - No

If yes, which language 🡪 _____________________

1. **Were you born in Canada?**
   - Yes
   - No

If no, what year did you arrive in Canada? ___________

1. **Do you identify as First Nations, Métis and/or Inuk/Inuit**?
   - Yes, First Nations
   - Yes, Métis
   - Yes, Inuk/Inuit
   - No
2. **In our society, people are often described by their race or racial background. These are not based in science, but our race may influence the way we are treated by individuals and institutions, and this may affect our health. Which category(ies) best describes you? Check all that apply:**

- Arab, Middle Eastern or West Asian
- Black
- East Asian
- Indigenous (First Nations, Metis, Inuk/Inuit)
  - Do you identify as Two-Spirit? Yes/No
- Latino/Latina/Latinx
- South Asian or Indo-Caribbean
- Southeast Asian
- White
- Another race category: ________________

1. **In general, do you experience any of the following due to a physical, mental, or emotional condition? *(select all that apply)***

- Difficulty seeing
- Difficulty hearing
- Difficulty walking or climbing
- Difficulty remembering or with concentration
- Difficulty with self-care
- Difficulty with communicating
- None of the above

1. **A) What was your sex assigned at birth? *(check one)***
   - Female
   - Male
   - Intersex
2. **B) What is your current gender identity? *(check one)***
   - Woman
   - Man
   - Transgender
   - Gender fluid or Gender nonbinary
   - Two-Spirit (Indigenous)
   - Another (Specify)__________
3. **Which best describes your sexual orientation?**
   - Heterosexual (“straight”, male/female relationships or two different binary genders)
   - Gay
   - Lesbian
   - Bisexual
   - Queer or Pansexual
   - Two-Spirit (Indigenous)
   - Another (Specify)_____________

**Social needs**

1. **What is the highest level of education you have completed?**
   - Some grade school
   - Completed grade school (grade 1-8)
   - Some high school
   - Completed high school (grade 9-12)
   - Trades Certificate/Diploma
   - Some college/university
   - College/university degree
   - Postgraduate degree
   - No formal schooling
2. **Do you have difficulty making ends meet at the end of the month?**
   - Yes
   - No
3. **In the last 12 months, did you avoid filling a prescription or do anything to make a prescription last longer *because of the cost*?**
   - Yes
   - No
   - Not Applicable
4. **a) What is your current housing?**
   - Own home
   - Rent
   - Staying with friends or relatives because you have no alternative [couch surfing]
   - Shelter
   - On the street
   - Other (Specify)_______________

**b) If you rent 🡪 Is your current housing social housing, subsidized housing, or rent-geared-to-income?**

- - Yes
  - No
  - Not Applicable

**c) If own home/rent 🡪 During the last 12 months, was there a time when you were not able to pay the mortgage or rent on time?**

- - Yes
  - No
  - Not Applicable

1. **a) Do you feel you have family or close friends who you can open up to?**

- Yes
- No

**b) Are you able to rely on them if you need help (e.g., transportation, emotional or financial assistance)?**

- Yes
- No
- Not Applicable

1. **In the past 12 months, did you avoid attending an important appointment because of the cost of transportation?**

- Yes
- No
- Not Applicable

1. **In the past 12 months, did you miss making a payment on your electric, gas or other utilities bills *because of cost*?**

- Yes
- No
- Not Applicable

**16. a)** **Are you employed in a casual, short-term or temporary position?**

- Yes
- No
- Not Applicable

b) **Do you feel fearful that you could be fired if you raise employment concerns?**

- Yes
- No
- Not Applicable

c) **Does your pay vary a lot from month to month?**

- Yes
- No
- Not Applicable

**References**

1. Davis VH, Rodger L, Pinto AD. Collection and Use of Social Determinants of Health Data in Inpatient General Internal Medicine Wards: A Scoping Review. *J Gen Intern Med*. 2022;38(2):480-489. doi:10.1007/s11606-022-07937-z

2. Davis VH, Dainty KN, Dhalla IA, Sheehan KA, Wong BM, Pinto AD. “Addressing the bigger picture”: A qualitative study of internal medicine patients’ perspectives on social needs data collection and use. *PLOS ONE*. 2023;18(6):e0285795. doi:10.1371/journal.pone.0285795

3. Adekoya I, Delahunty-Pike A, Howse D, et al. Screening for Poverty And Related Social determinants to improve Knowledge of and links to resources (SPARK): development and cognitive testing of a tool for primary care. Published online December 31, 2022:2022.12.30. doi:10.1101/2022.12.30.22283580
