# Supplementary material for: Perspectives on Using Artificial Intelligence to Derive Social Determinants of Health Data From Medical Records in Canada: Large Multijurisdictional Qualitative Study
Source: J Med Internet Res. 2025 Mar 6;27:e52244. doi: 10.2196/52244 (PMC11926464; doi:10.2196/52244)
Supplement: Multimedia Appendix 2 [file jmir_v27i1e52244_app2.docx]

**Appendix 2. Interview Questions Assessing Sociodemographic Data Collection using Artificial Intelligence**

12. Imagine our electronic medical record (EMR) could have a program running in the background that could determine your race, income, gender identity based on existing data in your EMR. So for example, you went to see your family doc and you were talking about your housing or employment situation and your doc made notes in your EMR. Then you had this software that could go through your EMR and identify or fill out your demographics based on existing data in your EMR. This information captured by the software will remain part of the secure medical record.

1. What are your thoughts on using computers to determine this information, rather than ask people one by one?
2. What are some of the benefits of this?
3. What are some of the concerns you have?
