# Supplementary material for: Perspectives on Using Artificial Intelligence to Derive Social Determinants of Health Data From Medical Records in Canada: Large Multijurisdictional Qualitative Study
Source: J Med Internet Res. 2025 Mar 6;27:e52244. doi: 10.2196/52244 (PMC11926464; doi:10.2196/52244)
Supplement: Multimedia Appendix 3 [file jmir_v27i1e52244_app3.docx]

**Appendix: Additional Representative Quotes**

| **Theme or sub-theme** | **Representative Quotes** |
| --- | --- |
| ***Artificial intelligence as the inevitable future: facilitating more efficient, accessible sociodemographic information and use*** | *“I mean it’s technology. It’s the world we’re living in; right, and it’s much better that way.” – 05_20 (Newfoundland and Labrador)*  *“I have no problem really you know because with the advancement; right, like you know and [Pause] that we will be moving forward; right, like the computers will be doing the stuff right for us.” – 01_103 (Ontario)*  *“I think that’s not avoidable in the future. [Laugh] So, I am okay with that…Because that’s where we’re going to, that’s where we are [Pause] heading to.” 03_10 (Saskatchewan)*  *“It sounds like the direction that the world is sort of going in because everything is technology based and there’s not a lot of human interaction like computers; technology is really [Pause] just taking over everything so it’s not a surprise that’s, those are my thoughts. It’s kind of like I saw this coming.” – 01_82 (Ontario)* |
| **Efficient, streamlined social determinants of health data** | *“The benefits of that is really quickly doing this whole thing as opposed to waiting for individuals to go through this ten-minute survey and multiplying that by a population of 30 million Canadians.” -01_13 (Ontario)*  *“Efficiency, time… It’s there right away. It’s just a lot more [of] a smooth and efficient way of doing things.” – 05_20 (Newfoundland and Labrador)* |
| **Artificial intelligence could overcome barriers to disclosure** | *“It’s good in the sense that it [would] remove the confusion individuals may have about certain questions…. If it’s a complicated enough question which I don’t usually have, I haven’t come across except probably the sexual orientation ones.” - 01_13 (Ontario)*  *“I mean I guess it does save people the time to fill out the questionnaires whereas because you don’t have to actually go through it and for people who are immigrants that struggle with like language [Pause] they wouldn’t have to fill it out and it would just be filled out for them automatically.” – 01_69 (Ontario)* |
| **Data use to improve health** | *“I think the…benefit is just being able to see like potential cases… for people that haven’t went to the doctor for a while. Being able to just see…their race and their current health. They might automatically [use this information to create] a warning…[saying that someone may have diabetes]…So, [their doctors in] the future…[could] help them easier.” – 03_08 (Saskatchewan)*  *“AI can [be used] if you want some [help with] decisions… but we are dealing with living people. We are not expecting any result from this. If you want [data saying] like these are ten males and twelve females and these are some literate peoples, these are some illiterate peoples….then you can use the AI but not for the one-to-one.” - 01_54 (Ontario)*  *“I mean if this software you know could come [Pause] up with resources you know like all of a sudden… I mean my doctor doesn’t know everything or where to find services and stuff so like if this software could, so, [participant name] came in and he was sad on this day and he expressed feelings of depression and anxiety so maybe then the software would be able to instantly find resources in the neighbourhood close by [Pause]…[Let’s say] you are experiencing housing concerns you know so right away there is a list of shelters or cheap housing.” - 01_92 (Ontario)* |
| ***Potential health care harms: distrust in artificial intelligence and public systems*** |  |
| **Artificial intelligence inaccuracies and impact on care** | *“First of all, misclassifications. It’s going to assume all sorts of things and I don’t think we need artificial intelligence handling these kinds of very intimate, personal items…You know you’re going to define my gender…I just think it won’t be accurate and I kind of find it insulting you know as a person.” -01_28 (Ontario)*  *“I feel like yeah, you’re just trying to [Pause] you know judge me and like trying to get okay, this could, like I don’t know how come it, this would work but trying to assume that my religion is X and trying to assume that okay, this like I work as a data analyst but I don’t really feel that you can 100% assume like someone’s religion or someone’s race cause yeah, I mean you for example, you might find many people who are coming from the Middle East or Africa or well Asia but they are so westernized so okay, like if you will try to estimate their race maybe it will not be accurate.” -03_11 (Saskatchewan)*  *“I mean [if] any answer for that particular patient is wrong then it may impact [their] prognosis and diagnosis as well.” – 01_54 (Ontario)*  *“Computers mostly work off like algorithms and patterns and that’s not really good for people cause every individual is different so if you tried to group you know race and ethnicity and you try to talk about them specifically at the end of the day it’s maybe the majority but it’s never 100% of the people who share the same experience.” – 01_11 (Ontario)*  *“I mean I would trust [AI] cause sometimes [people] don’t even make sense…I would be trusting of it but then it’s also like concerning for…other people because sometimes like…the computer can make mistakes… I guess like it’s worth it given like the circumstances that we’re in too so yeah, I don’t see why not… The benefits were like you don’t have a person that might be misdiagnosing you.” – 01_138 (Ontario)*  *“I think one of the [Pause] difficulties would be [Pause] making sure that the doctor was [Pause] acknowledging the proper language for that software to understand and that the patient was explaining it properly as well… I think if [the doctor relies] more on technology to do that sometimes I feel that doctors may [Pause] allow those kinds of updates [on their patients’ social situation] to kind of slip through the cracks and it may not be as up to date potentially as a conversation with a physician.” – 01_48 (Ontario)*  *“Plus some of those things change overtime right like someone who's transgender may have written man and then five years later they're woman or transgender so [Pause] well then again maybe the AI updates that stuff but anyway, long story short I prefer to be asked about it and my responses recorded versus kind of like I don't know data aggregate.” – 01_02 (Ontario)*  *“I think it would probably lack depth because [Pause] if that’s going on information that’s just ten years old I think the climate has changed around some of these things, especially around race and [Pause] gender expression…Extracting from old files may extract data that’s not really truly representative of the client’s current status.” - 05_12 (Newfoundland and Labrador)*  *“Well that information I guess can change; right, so you would want to make sure it’s up to date because maybe things, maybe financially your situation changes in a week or two between doctor and specialist visits or things like that, I guess.” – 01_81 (Ontario)* |
| **Privacy and security** | *“So any electronic data that’s maintained has to be secured enough…because data is part of the future--so every private companies or insurance company they get a hold on it then…the whole profile could change their metric depending on this information especially health care I have seen.” - 01_102 (Ontario)*  *“I guess [Pause] I guess the biggest and only concern would be privacy data breached through like a third-party hack-in or something cause [Pause] we have had our medical records hacked here in Saskatchewan and Alberta.” -03_24 (Saskatchewan)*  *“I’m sorry to say this but the health care industry is lagging when it comes to data and all of that. It lags behind tech generally behind finance so the people who are doing this need to be brought up to speed about all of the multiple issues around data privacy and cyber security…the infrastructure and the environment in which you are operating is not equipped to protect the data you are collecting.” - 01_13 (Ontario)*  *“Well, again I think that the privacy…has to be the number one issue that guides every aspect of this and…the patient has to be the one who decides what is transferred and to where is it transferred…My fear is [Pause] that the system is too busy and messy and disorganized [Pause] to handle it appropriately and that mistakes will be made all over the place… It’s frightening what computers are being programmed to do without any [Pause] accountability… I think if the average Joe and Jane on the street knew [Pause] people would be very upset about it.... We go in, we answer a question and it’s like [Pause] no big deal and all of a sudden [Pause] something really private is [Pause] landing on somebody’s desk that is totally irrelevant.” – 04_21 (Manitoba)*  *“I think that’s a huge invasion of privacy…When I’m talking to my doctor I don’t expect him to be, like I expect him to be taking notes but relevant notes. I don’t see how taking notes that I work part-time would be [Pause] relevant and then having it [Pause] like [Pause] without my consent being put on the SPARK Tool I think that’s an invasion of privacy…[I’m concerned] that it would be leaked. That that data would be-collected on my behalf and then I don’t know sold off.” – 01_80 (Ontario)* |
| **Data misuse and discrimination** | **“***… [Or, for example], Latinos’ hypertension: it will be useful for me to say I’m Latino and then they’ll be like oh, let’s take care of this … but [Pause] at the same time it can go the other way… With AI they were able to like detect where… it was more likely to [have crimes happen] and they were trying to [hire more] police in those sectors--but I think…that ends up profiling folks.” – 01_07 (Ontario)*  *“Well, and it’s based on a whole lot of basically stereotyping groups so saying oh, well I am racialized and low-income therefore these things must be true [Pause] and I’m not at all comfortable with, cause it’s, the whole system relies on those stereotypes and assumptions [Pause] and I don’t think that’s a good way to do it….If you’re going to extrapolate based on survey responses [Pause] then you’re going to be, you have to base that on broad generalizations and that’s just not going to provide accurate information about the specific person. It works on a population level not on an individual level so I wouldn’t want anything on my chart based on population level statistics.” – 04_17 (Manitoba)* |
| ***Loss of the human touch: preference for provider relationships and individualized care*** | *“I mean I don’t know the computer. I can’t ask any questions, I can’t speak to someone and share concerns. I don’t necessarily know why it’s being collected. There is no like interaction there... If I want to complain to someone, how would I complain [to] a computer?” – 01_26 (Ontario)*  *“I think it’s somewhat for a medical field, especially people struggling with mental health, it can be dehumanizing so these people feeling isolation could feel more isolation through just being dealt with by a machine.” – 01_125 (Ontario)*  *“I think its de-personalizing encounters with the doctor to the point [Pause] where [Pause] you know if the goal is to cut down the time that you see the doctor I think most of us [Pause] don’t see, get to have [Pause] big in-depth encounters with our doctors anyway.” - 04_20 (Manitoba)*  *“[I can imagine] a fluid conversation where I actually get to build rapport with [my health care provider], I get to feel connected to my health care provider and that they actually understand me and they’re not just looking at me through this piece of, through the survey tool…And then sure, having a program or AI running in the background that is recording the conversation and then accept the key pieces of information that it needs to know and inserts them into the chart.” - 01_09 (Ontario)*  *“I think that would just be a lot more generalizing than making it all about statistics when it should really be about [Pause] trying to get what’s best for people and people have concerns that they can’t you know choose from a selection of things that they need to type out and voice.” 01_36 (Ontario)*  *“I think that was probably my concern is just over generalizing it and not really looking at the individual cause [Pause] there’s so many different [Pause] different factors amongst different groups if you really pay attention…so to [Pause] for a broad perspective sure, I think artificial intelligence could be very beneficial but [Pause] also making sure that we are leaving room and we’re educating the nurses and the doctors to not just only use that [Pause] but to continue to look at the individual.” 03_03 (Saskatchewan)*  *“Once you’re seeing that patient you shouldn’t be just looking at the demographic tool and that be all because [Pause] somebody who is [Pause] Indigenous could have a different perspective with the health care setting, they could have a different life. One of my good girlfriends she’s Indigenous [Pause] she’s currently in med school. I have other Indigenous friends who are living in the homeless shelter so to go ahead and to only be placing people of different groups into different [Pause] categories I think would be very dangerous because then you wouldn’t be really looking at how you can actually serve that person and help that individual.” 03_03 (Saskatchewan)*  *“I think what we’re lacking these days is physical, emotional connection to people. I think that a questionnaire like this again being filled out with your provider, oh, can make such a world of difference, I think.”* 01_77 *(Ontario)* |
| ***Consent is critical: strong safeguards are needed to protect patients’ data and trust*** | *“I think people might see it as a breach of trust because they’re not being asked directly so they’re not… giving consent for this information to be collected and stored…I would be a little bit weirded out by it like I would [rather] just be asked directly and be told that this information is going to be stored on my file… I would want that full transparency and I think people deserve that transparency.”- 01_78 (Ontario)*  *“It would be great if people know that this information is being collected and just explain to them why…No one wants to be caught off guard that that information is being collected [but they] were unaware and didn’t give consent to it.” – 01_38 (Ontario)*  *“Yeah, I don’t have a problem with that. If somebody has consented to provide that information and explain, and it’s explained to them how it can be used I don’t have a problem with that but again it’s informed consent.” - 04_03 (Manitoba)* |
| **Data access and verification** | *“I think that I would need it to be [Pause] like [Pause] like tentative… the software would be able to like put a question mark beside things that like [Pause] I thought was relevant but might actually not be relevant so rather than the software being deterministic the software being like tentative…and then my doctor next time can maybe check-in on it and it’ll be easier.” - 01_09 (Ontario)*  *“Well, I’d want it to be available to me upon request…So [Pause] I’d want to have the ability to make sure my health care information that was being accessed or used to evaluate me was accurate…Have the option at least.” – 05_13 (Newfoundland and Labrador)*  *“I feel like it might have a few inaccuracies but [Pause] if they can identify that it sounds pretty cool. I would just want to double check [what it identifies me as] cause I feel like it might like [Pause] I don’t know it might identify me as something else…It sounds, maybe like you know maybe like, because right now I’m with my parents and then all of a sudden, I go out on my own and like end up living on the street when it identified me as being in a high-income household and like just being able to change that information readily but- like cause I think like it’s hard like nobody stays in the same places. Everyone changes so that’s the only concern I have but other than that no.” - 01_45 (Ontario)* |
